# DNX-2401, an Oncolytic Virus, for the Treatment of Newly Diagnosed Diffuse Intrinsic Pontine Gliomas: A Case Report

**DOI:** 10.3389/fonc.2018.00061

**Published:** 2018-03-12

**Authors:** Sonia Tejada, Ricardo Díez-Valle, Pablo D. Domínguez, Ana Patiño-García, Marisol González-Huarriz, Juan Fueyo, Cande Gomez-Manzano, Miguel Angel Idoate, Joanna Peterkin, Marta M. Alonso

**Affiliations:** ^1^Department of Neurosurgery, University Hospital of Navarra, Pamplona, Spain; ^2^The Health Research Institute of Navarra (IDISNA), Pamplona, Spain; ^3^Program in Solid Tumors and Biomarkers, Foundation for the Applied Medical Research, Pamplona, Spain; ^4^Department of Radiology, University Hospital of Navarra, Pamplona, Spain; ^5^Department of Pediatrics, University Hospital of Navarra, Pamplona, Spain; ^6^Brain Tumor Center, The University of Texas MD Anderson Cancer Center, Houston, TX, United States; ^7^Department of Pathology, University Hospital of Navarra, Pamplona, Spain; ^8^DNAtrix, Inc., Houston, TX, United States

**Keywords:** diffuse intrinsic pontine gliomas, phase I clinical trial, oncolytic virus, delta-24-RGD, DNX-2401, intratumoral, MEMS cannula, biopsy

## Abstract

Diffuse intrinsic pontine gliomas (DIPGs) are aggressive glial brain tumors that primarily affect children, for which there is no curative treatment. Median overall survival is only one year. Currently, the scientific focus is on expanding the knowledge base of the molecular biology of DIPG, and identifying effective therapies. Oncolytic adenovirus DNX-2401 is a replication-competent, genetically modified virus capable of infecting and killing glioma cells, and stimulating an anti-tumor immune response. Clinical trials evaluating intratumoral DNX-2401 in adults with recurrent glioblastoma have demonstrated that the virus has a favorable safety profile and can prolong survival. Subsequently, these results have encouraged the transition of this biologically active therapy from adults into the pediatric population. To this aim, we have designed a clinical Phase I trial for newly diagnosed pediatric DIPG to investigate the feasibility, safety, and preliminary efficacy of delivering DNX-2401 into tumors within the pons following biopsy. This case report presents a pediatric patient enrolled in this ongoing Phase I trial for children and adolescents with newly diagnosed DIPG. The case involves an 8-year-old female patient with radiologically diagnosed DIPG who underwent stereotactic tumor biopsy immediately followed by intratumoral DNX-2401 in the same biopsy track. Because there were no safety concerns or new neurological deficits, the patient was discharged 3 days after the procedures. To our knowledge, this is the first report of intratumoral DNX-2401 for a patient with DIPG in a clinical trial. We plan to demonstrate that intratumoral delivery of an oncolytic virus following tumor biopsy for pediatric patients with DIPG is a novel and feasible approach and that DNX-2401 represents an innovative treatment for the disease.

## Introduction

Diffuse intrinsic pontine gliomas (DIPGs) are brain tumors located in the pons, the diagnosis of which is based on magnetic resonance imaging (MRI). The 2016 World Health Organization (WHO) classification of Central Nervous System tumors regards these tumors as *diffuse midline gliomas H3 K27M-mutants* (1). There is no biopsy requirement (2) to confirm tumor histopathology prior to initiating currently available treatment which is radiotherapy, with or without chemotherapy (3). However, stereotactic biopsy for pediatric brainstem tumors is associated with a low risk of complications and could be essential for individualizing treatment based on molecular profiling (4). The prognosis for these young patients is dire, with a median overall survival of only 12 months (5).

The lack of effective treatments for DIPG has propelled the field into developing alternative therapeutic strategies that includes biological agents. Oncolytic adenoviruses modified to replicate in, and selectively destroy, tumor cells represents a promising new treatment approach for DIPG. DNX-2401 (formerly Delta-24-RGD) is a replication-competent adenovirus modified to enhance tropism to glioma cells and replicate in cancer cells that have a defective Rb pathway. The virus has already demonstrated safety and efficacy in animal models of gliomas (6, 7), adults with recurrent glioblastoma (8), and animal models of DIPG (9).

We have designed an open-label, dose-escalating clinical trial to determine the safety and efficacy (tumor response and survival), of single dose intratumoral DNX-2401 following biopsy for pediatric patients with newly diagnosed DIPG (10). The starting dose of DNX-2401 at 1 × 10^10^ viral particles (vp) is lower than that used in adults (5 × 10^10^) but may be increased to 5 × 10^10^ if no toxicity is reported. Secondary aims are to collect tissue samples and evaluate possible synergy of DNX-2401 with radiotherapy and chemotherapy.

## Case Presentation

The patient, an otherwise healthy 8-year-old female who presented with vomiting and a 4-month history of gait disturbance, was initially evaluated for food intolerance. Following complaints of diplopia and headache, MRI without gadolinium (gd) enhancement revealed an infiltrating tumor across the entire length of the pons that was hypointense on T1 and hyperintense on T2 sequences. The lesion was diagnosed as DIPG (10). The patient met all clinical trial eligibility criteria (EudraCT: 2016-001577-33), and her parents provided written informed consent for their daughter to participate in the study.

**Figure 1:**
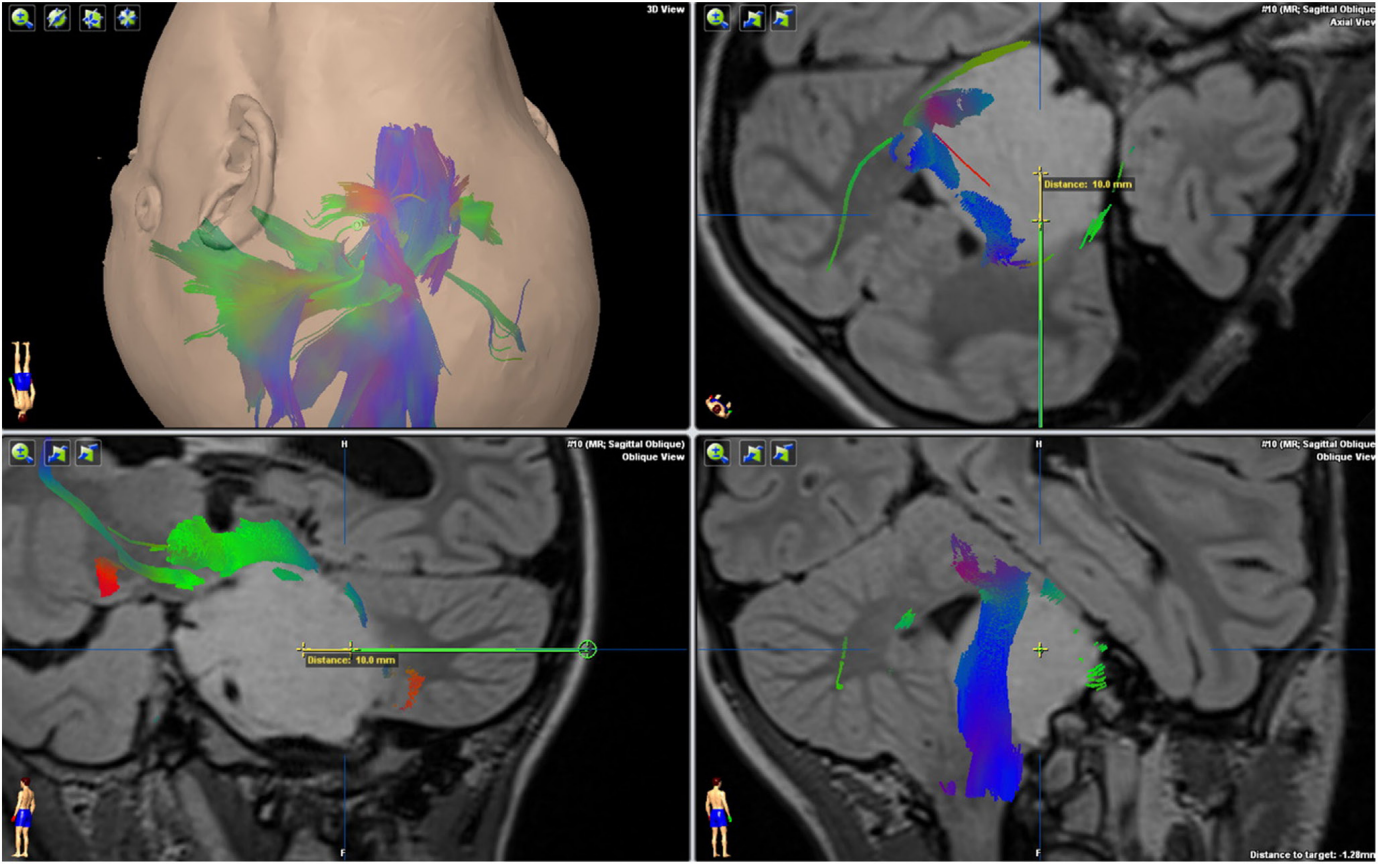
Magnetic resonance imaging T2 FLAIR 3D sequence, with DTI tractography fusion showing the relationship between the tumor and major brain tracts. The green trajectory of the biopsy avoids the corticospinal tract ending in the biopsy target. The yellow segment notes the extra depth (10 mm) of the infusion cannula tip.

A transcerebellar, transpeduncular frameless navigated biopsy was performed through a suboccipital entry point, using the Brainlab VarioGuide navigation system (Figure 1). This navigated stereotactic system has proven accuracy for brainstem biopsies (11). Stereotactic guidance to localize the biopsy target was based on a preoperative immediate 3T MRI machine (Skyra, Siemens, Erlangen, Germany). Following withdrawal of the biopsy needle, a cannula specially designed for the delivery of agents into the brain (Alcyone MEMS cannula) was introduced along the same trajectory; however, 10 mm deeper than the tip of the biopsy needle. The MEMS cannula is a device with two fused microtubes or channels that permit the administration of two independent fluids once inserted into the tissue.

For the patient, one channel was used to infuse 150 μL of gd (2 mM) at 0.9 mL/h (10 min) while the second channel was used to deliver 1 × 10^10^ vp in 1 mL of DNX-2401 into the tumor at 0.9 mL/h (67 min). The gd was subsequently pushed out of the tumor tissue by the virus as it was infused from the second channel.

**Figure 2:**
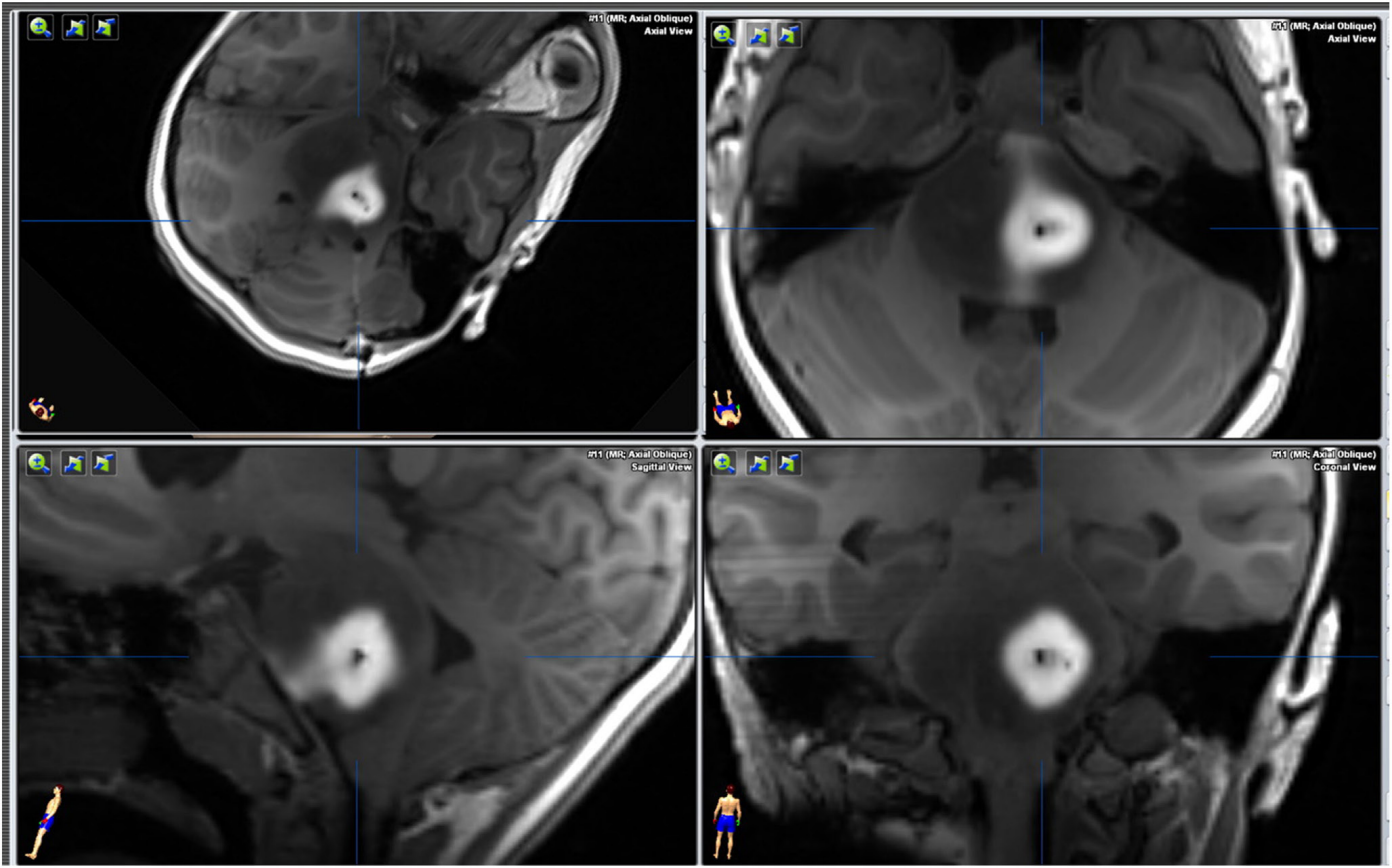
Magnetic resonance imaging T1 GE 3D axial sequence without intravenous contrast, immediately after virus infusion. The intraparenchymal injection of gadolinium before infusing the virus with the MEMS cannula (this cannula has two independent channels) is pushed out from the tumor as the virus is infused from a second channel.

**Figure 3:**
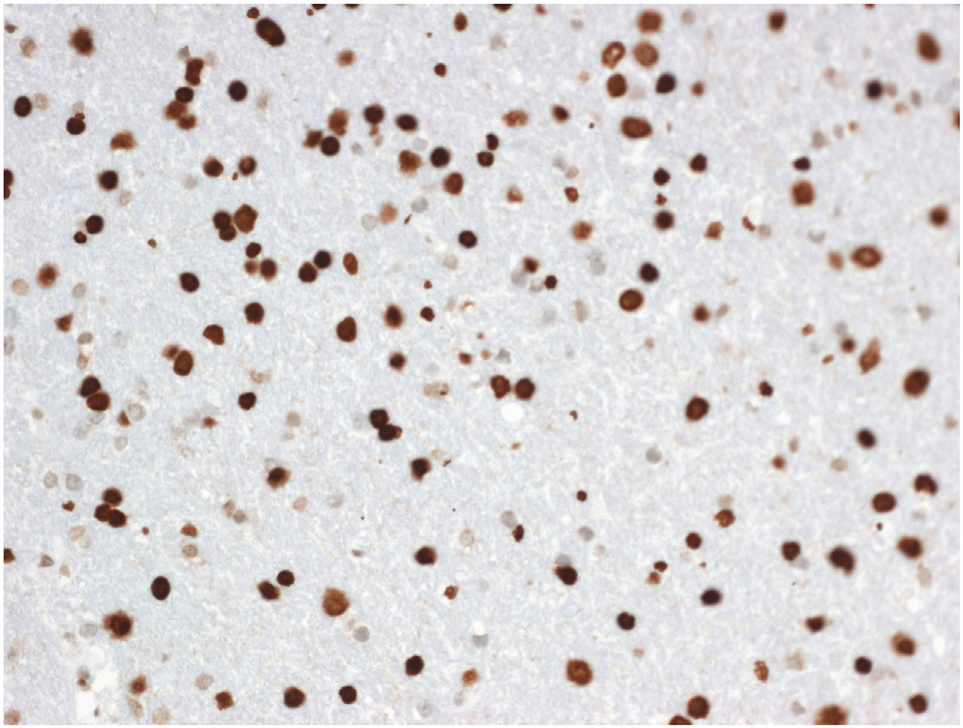
Diffuse midline glioma showing strong nuclear H3K27M mutant protein (immunohistochemistry, X200).

An intraoperative MRI was performed immediately after DNX-2401 administration that showed no biopsy complications and gd diffusion through the tumor thereby confirming successful targeted delivery of the virus (Figure 2). The patient tolerated the procedures and was discharged to home from the hospital in 72 h. The pathologist later classified the tumor as a WHO grade II glioma with a H3.3 K27M mutation that was determined by Sanger sequencing (Figure 3). The patient started radiotherapy 2 weeks after the procedure without difficulty.

## Discussion

During the last few decades, DIPG has been considered an inoperable tumor because there have been no benefits gained from biopsy or resection surgery (12, 13). However, there has been a resurgence in recent years of the need for performing a biopsy for different reasons: inconclusive images on MRI for atypical lesions (14), the design of clinical trials for DIPG (15), and a significant need to learn more about tumor biology and genetics (16, 17).

Although there is a low risk of complications from biopsy (18), the possibility still remains making the procedure difficult to justify outside a clinical trial setting (2). On the other hand, analyzing biopsied tissue will advance our fundamental knowledge of this tumor with the goal of developing new treatments (19) taking intrinsic characteristics such as molecular aberrations and the tumor microenvironment into account. This information has the potential to alter the diagnostic perspective and treatment options to achieve the best patient outcome. Furthermore, tissue analysis will aid the development of reliable transgenic animal models of DIPG (20).

We believe that tumor biopsy followed by targeted therapy in a single surgical session seems to offer the best balance. In this way, the risks of biopsy are offset by the potential therapeutic benefits of novel therapeutics, such as DNX-2401.

DNX-2401 has shown compelling efficacy in animal models (21, 22). The drug has also been assessed in adults with recurrent glioblastoma in several ethics committee-approved clinical trials at different investigative centers including our hospital, the University Hospital of Navarra (EUDRACT number: 2011-005935-21). To date, no severe or greater virus-related toxicity has been observed (i.e., no ≥grade 3 in severity per the CTCAE v4.0 toxicity criteria) at a dose of 3 × 10^10^ vp. Median survival has been improved over standard of care including surgery, with some patients surviving three or more years.

DNX-2401 was successfully delivered into DIPG of the first study patient *via* the MEMS cannula fabricated to deliver fluid into the brain using micro-electro-mechanical systems technology that avoids backflow (23) and confirmed by MRI demonstrating the gd halo. Precise administration into the target lesion increases the potential for an anti-tumor response. In addition, while animal data suggest DNX-2401 followed by radiation, standard therapy for DIPG, improves outcome compared with radiation alone, all patients receive both treatments. As suggested by O’Cathail et al., two treatments given sequentially could be synergistic, leading to a more robust anti-tumor response (24). This hypothesis also provides a rationale for infusing the virus as first-line therapy before radiation in treatment-naive patients.

Overall, the study patient did not experience any procedure-related safety issues or virus toxicity during the first 4 weeks following biopsy and virus infusion. Longer term follow-up and the enrollment of additional pediatric patients are necessary to confirm these preliminary results.

## Conclusion

This case report describes the first time an oncolytic adenovirus has been injected into DIPG of a pediatric patient in the context of a clinical investigation: a “Phase I trial of DNX-2401 for DIPG for newly diagnosed pediatric patients.” Early data generated from this patient’s participation in the trial demonstrate biopsy and virus delivered during the same surgical session are both feasible. Additionally, DNX-2401 was well tolerated during the first weeks so that the patient was able to begin radiotherapy 2 weeks later.

We believe the information obtained from this trial could advance the understanding of this devastating disease and facilitate the development of new treatment strategies to alter the current treatment paradigm for DIPG.

## Ethics Statement

Before initiating the trial, the protocol and all required documents were reviewed and approved by the ethics committee (CEIC) from the Government of Navarra (D24-DIPG) and The Spanish Agency of Medicines and Health Products (AEMPs, Agencia Española del Medicamento y de Productos Sanitarios) Eudra nr 2016-001577-33. Patients younger than 12 years old are not required to sign the informed consent form. Instead, they receive the information by listening to the agreement regarding the treatment, and their capacity to make an informed decision will be confirmed. The patients’ parents will then sign the informed consent. Patients older than 12 years old can sign an informed consent along with their parent’s signature, and they will receive information according to their comprehension level. Written parental consent was provided by the parents on a consent form approved by the CEIC that described the trial and contained sufficient information to make an informed decision about their daughter’s participation in the trial. The designated investigator or research professional obtaining consent also signed the form. Additional written informed consent was obtained from the parents for the publication of this case report.

## Author Contributions

MA, ST-S, RD-V, JF, and CG-M designed the study. ST-S and RD-V perform surgery and virus delivery. AP and MG-H were in charge of sample collection. AP, MA, and MG-H performed and analyze the molecular study. PD performed and analyze images. ST-S, RD-V, JP, and MA wrote the manuscript. All the authors revised and approved the manuscript. MI performed and analyzed tissue samples, providing the figure and the legend.

## Conflict of Interest Statement

The authors declare that the research was conducted in the absence of any commercial or financial relationships that could be construed as a potential conflict of interest.
